# The effect of culture media on large-scale expansion and characteristic of adipose tissue-derived mesenchymal stromal cells

**DOI:** 10.1186/s13287-019-1331-9

**Published:** 2019-08-05

**Authors:** Justyna Czapla, Sybilla Matuszczak, Klaudia Kulik, Ewa Wiśniewska, Ewelina Pilny, Magdalena Jarosz-Biej, Ryszard Smolarczyk, Tomasz Sirek, Michał Oskar Zembala, Marian Zembala, Stanisław Szala, Tomasz Cichoń

**Affiliations:** 1 Center for Translational Research and Molecular Biology of Cancer, Maria Skłodowska-Curie Memorial Cancer Center and Institute of Oncology, Gliwice Branch, Wybrzeże Armii Krajowej 15 Street, 44-102 Gliwice, Poland; 2 Kardio-Med Silesia, Maria Skłodowska-Curie 10C Street, 41-800 Zabrze, Poland; 3 Hospital for Minimally Invasive and Reconstructive Surgery, Armii Krajowej 180 Street, 43-316 Bielsko-Biała, Poland; 4 Department of Cardiac Surgery and Transplantology, Silesian Centre for Heart Diseases, Maria Curie Skłodowska 9 Street, 41-800 Zabrze, Poland; 5 Department of Cardiac, Vascular and Endovascular Surgery and Transplantology, School of Medicine with the Division of Dentistry, Silesian University of Medicine, Maria Curie Skłodowska 9 Street, 41-800 Zabrze, Poland

**Keywords:** Adipose tissue-derived mesenchymal stromal cells, Advanced therapy medicinal products, Human platelet lysate, Interleukin-6

## Abstract

**Background:**

Adipose tissue-derived mesenchymal stromal cells (ASCs) have been shown to exhibit some promising properties of their use in regenerative medicine as advanced therapy medicinal products (ATMP). However, different sources of their origin, methods of isolation, and expansion procedures cause the laboratory and clinical results difficult to compare.

**Methods:**

ASCs were isolated from lipoaspirates and cultured in three different medium formulations: αMEM and DMEM as a basal medium supplemented with 10% of human platelet lysate (hPL) and DMEM supplemented with 20% fetal bovine serum (FBS) and bFGF as a gold standard medium. Subsequently, the impact of culture media on ASCs growth kinetics, their morphology and immunophenotype, ability to differentiate, clonogenic potential, and secretion profile was evaluated.

**Results:**

All cultured ASCs lines showed similar morphology and similar clonogenic potential and have the ability to differentiate into three lines: adipocytes, osteoblasts, and chondroblasts. The immunophenotype of all cultured ASCs was consistent with the guidelines of the International Society for Cell Therapy (ISCT) allowing to define cells as mesenchymal stromal cell (MSC) (≥ 95% CD105, CD73, CD90 and ≤ 2% CD45, CD34, CD14, CD19, HLA-DR). The immunophenotype stabilized after the second passage and did not differ between ASCs cultured in different conditions. The exception was the ASCs grown in the presence of FBS and bFGF, which expressed CD146 antigens. The secretion profile of ASCs cultured in different media was similar. The main secreted cytokine was IL-6, and its level was donor-specific. However, we observed a strong influence of the medium formulation on ASCs growth kinetics. The proliferation rate of ASCs in medium supplemented with hPL was the highest.

**Conclusions:**

Culture media that do not contain animal-derived antigens (xeno-free) can be used to culture cells defined as MSC. Xeno-free medium is a safe alternative for the production of clinical-grade MSC as an advanced therapy medicinal product. Additionally, in such culture conditions, MSC can be easily expanded in accordance with the Good Manufacturing Process (GMP) requirements to a desired amount of cells for clinical applications.

## Background

Mesenchymal stromal cells (MSC) have been isolated from various tissues and organs throughout the human body [1]. The exceptional ease of their isolation and ex vivo expansion additional to their ascribed regeneration potential led to the extremely broad application of MSC in a clinical trial [2] and subsequently led them to clinical medicine as advanced therapy medicinal products (ATMP) [3, 4]. However, many concerns and controversies arising from their clinical application in the so-called cell therapy are still not solved [5, 6]. The major issue is the heterogeneity of MSC population [7]. Despite meeting all the criteria of the International Society for Cellular Therapy (ISCT) [8], namely (1) plastic adherence in standard culture conditions, (2) defined expression of specific markers (CD73, CD90, CD105) along with lack of expression of hematopoietic markers (CD14, CD34, CD45, HLA-DR) and (3) differentiation potential into adipocytes, osteoblasts, and chondroblasts in vitro, MSC may differ in secretome and/or transcriptome profile depending on the donor of the tissue or the site of collection [5, 9, 10]. An additional drawback is the isolation and expansion protocols varying between laboratories. Different medium formulations used to culture MSC, especially animal-derived supplements used in MSC manufacturing, may lead to clinical complication and data misinterpretation. Accordingly, to consider MSC as an ATMP, precise thorough characteristics, large-scale quality, and relatively low-cost production of clinical-grade MSC need to be developed in xeno-free media.

In the study, MSC derived from human adipose tissues (in the paper, ASCs acronym will be used [11]) obtained by liposuction procedure were cultured in three different medium formulations: (1) αMEM supplemented with 10% human platelet lysate (hPL), (2) DMEM supplemented with 10% hPL, and (3) DMEM supplemented with 20% fetal bovine serum (FBS) and basic fibroblast growth factor (bFGF). Subsequently, we evaluated the effect of culture media on ASCs growth kinetics, their morphology and phenotype, ability to differentiate, clonogenic potential, and secretome profile.

No differences were found in the morphology, phenotype, differentiation potential, ability to clone forming, and secretome profile in all cultured ASCs. However, secretion of the most abundant cytokine (IL-6) was donor-specific. The growth kinetics was affected by medium formulation. The highest proliferation rate was observed in ASCs cultured in αMEM supplemented with 10% hPL medium.

## Methods

### Biological material used in the study

The material used in the study comprised of liposuction aspirates (*n* = 6) obtained from the Hospital for Minimally Invasive and Reconstructive Surgery in Bielsko-Biała. All female donors (mean age = 45 (range from 27 to 60 years old), BMI = 24.68 ± 2.45) undergo tumescent liposuction under general anesthesia performed by the same plastic surgeon. The area of liposuction in all cases was the abdomen. The donor site was previously infiltrated with Klein solution (1000 ml normal saline, 50 ml 1% lignocaine, 1 ml 1:1000 epinephrine, 12.5 ml sodium bicarbonate) and left for 10 min. Liposuction was performed using 3.0-mm 3-hole blunt tip cannula under low pressure (below 0.5 bar). Aspirate was collected directly to a sterile container. The experiments were performed in accordance with the Declaration of Helsinki, with the approval of the Institutional Review Board and Bioethical Committee (KB/430-62/13). All patients provided written informed consent for the collection of liposuction aspirates and subsequent analysis.

### ASCs isolation and culture

The obtained liposuction aspirates were washed with PBS^−^ (Gibco BRL, Paisley, UK) supplemented with 1% FBS (Gibco) until the remaining blood was removed. Fat tissue was digested with collagenase NB4 solution (0.39 U/ml, Serva Electrophoresis, Heidelberg, Germany) for 1 h at 37 °C. After digestion, the cell suspension was filtered through 70-μm and 40-μm strainers. The suspension of single cells (16.6 × 10^3^ cells/cm^2^) was seeded on plastic culture plates in 3 different culture media: (1) αMEM medium (Macopharma, Tourcoing, France), supplemented with 10% human platelet lysate (Macopharma); heparin (2 U/ml, Polfa, Warszawa, Poland); and penicillin-streptomycin (Sigma Aldrich, St. Louis, MO, USA); (2) DMEM supplemented with 10% human platelet lysate (Macopharma), heparin (2 U/ml, Polfa), and penicillin-streptomycin (Sigma Aldrich); and (3) DMEM supplemented with 20% FBS (Gibco), basic fibroblast growth factor (bFGF, 10 ng/ml, eBioscience, San Diego, CA, USA), and penicillin-streptomycin (Sigma Aldrich) [12]. After 72 h, the plates were rinsed with a fresh culture medium. Cell cultures were grown in an incubator under standard culture conditions (at 37 °C in 5% CO_2_). Cells were passaged at 80–90% confluency using 0.25% trypsin solution (Sigma Aldrich). ASCs from passage 2 were used for subsequent experiments.

### ASCs immunophenotypic analysis and differentiation analysis

The phenotype of ASCs was determined directly after cell isolation and in the subsequent passages (from 0 to 4 passages) by flow cytometer (BD FACSCanto™, BD, Franklin Lakes, NJ, USA) using specific antibodies. The cells were incubated with appropriate antibodies directed against the following human antigens: CD29-FITC (eBioscience), CD105-APC, CD73-PE, CD90-PE-Cy7, CD44-FITC, CD34-PE-Cy7, CD31-FITC, CD146-PE, KDR-PE, LIN-FITC, CD45-PE, and HLA-DR-PE-Cy7 (BD Biosciences, San Jose, CA, USA) or isotype-matched control antibodies were used. 7-Amino-actinomycin D (7AAD) (eBioscience) was used to stain non-viable cells just before running the flow analysis.

The Human Mesenchymal Stem Cell Functional Identification Kit (R&D Systems, Minneapolis, USA) was used to differentiate ASCs into adipocytes, osteoblasts, and chondroblasts. The procedure was performed in accordance with the manufacturer’s instructions. Adipocytes were stained with FABP4 antibody (Abcam, Cambridge, UK), and a secondary antibody linked to FITC fluorochrome (Fluorescent anti-rabbit IgG Kit, Vector Laboratories, Burlingame, CA, USA). Fluorescence imaging of stained cells was performed using a LSM710 confocal microscope (Carl Zeiss Microscopy GmbH). The ability of the cells to differentiate into chondroblasts and osteoblasts was assessed by histochemical staining using Safranin O and Alizarin Red, respectively (Sigma Aldrich). The stained cells were visualized using an Eclipse 80i microscope (Nikon Instruments Inc., Melville, NY, USA).

### Assessment of ASCs clonogenic potential

ASCs from 3 different culture media were treated with 0.25% trypsin solution and placed in a corresponding culture medium in plates at a density of 1 cell/3 cm^2^. ASCs cultures were grown in an incubator under standard culture conditions. After 10 days, the plates were washed twice with PBS^−^ buffer and stained with Giemsa solution for 15 min at room temperature. Arising clones were visualized using Eclipse 80i microscope. Clones consisting of more than 50 cells were counted. Clonogenic potential of cells was calculated using the formula:

$$ \frac{\mathrm{the}\ \mathrm{number}\ \mathrm{of}\ \mathrm{created}\ \mathrm{clones}}{\mathrm{the}\ \mathrm{initial}\ \mathrm{number}\ \mathrm{of}\ \mathrm{cells}}\times 100\% $$

### Analysis of ASCs secretome

ASCs (second passage) were plated at high density in a complete medium. On the next day of the culture, the medium was replaced with a medium without serum or hPL. Following 48 h, the medium was collected. The type and quantity of cytokines and growth factors secreted by ASCs were assessed using the Human Cytokine Antibody Array C5 kit (RayBiotech, Norcross, GA, USA). The analysis was conducted in accordance with the manufacturer’s instructions. Densitometry calculations were performed using an ImageJ 1.48y software (NIH) to analyze the quantity of cytokines and growth factors secreted by ASCs.

### The determination of interleukin 6 secreted by ASCs in vitro

The concentration of IL-6 secreted by ASCs was determined by ELISA (eBioscience) in accordance with the manufacturer’s instruction. The total protein in the homogenates was determined by the Bradford method.

## Results

**Fig. 1:**
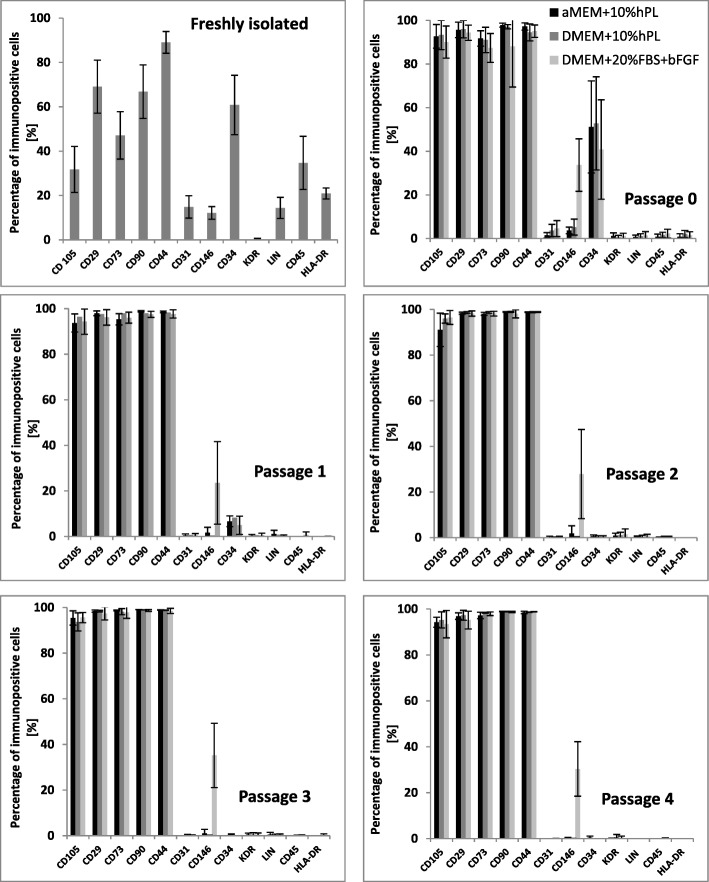
ASCs surface markers expression. The plots show the phenotype of ASCs obtained directly after ASCs isolation (before seeding on plates) and in the subsequent passages. Passage “0” indicates cells seeded after isolation that were trypsinizated when obtained 80–90% confluency. The cell phenotype stabilizes from passage 2 (expression of surface markers according to the guidelines of ISCT). The graphs summarized the surface marker expression on cells obtained from six isolation from different donors (*n* = 6)

**Fig. 2:**
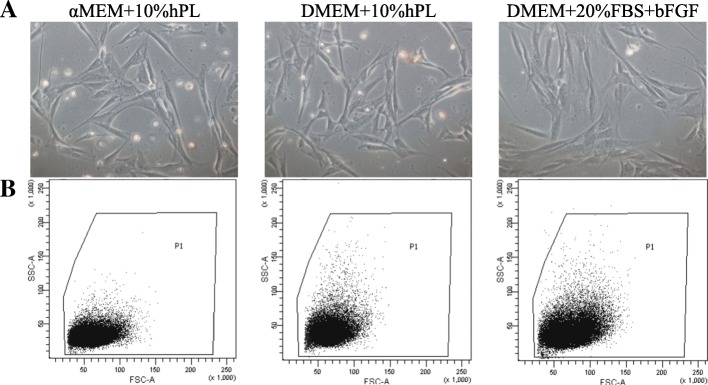
The morphology of ASCs cultured in three different medium formulations. **a** Phase-contrast images of ASCs at passage 1 (40% of confluency, 3–5 days of culture) parallel cultivated in either FBS- or hPL-supplemented basal medium. **b** Slight differences in the morphology between ASCs cultured in three different medium formulations were confirmed by flow cytometry gating of forward vs side scatter (FSC vs SSC). ASCs cultured in media supplemented with hPL were smaller and exhibited less complexity (granularity)

Adipose tissue collected by liposuction was obtained from the Hospital for Minimally Invasive and Reconstructive Surgery, Bielsko-Biała, Poland. For the purpose of this paper, only the abdominal fat was used. We isolated cells from six healthy female donors under 60 years. After isolation, before seeding on plates, cells were immunophenotyped by flow cytometry using the specific antibodies. Flow cytometry analysis showed that freshly isolated adipose-derived cells, following collagenase digestion, did not possess phenotype of mesenchymal stromal/stem cells according to the guidelines of the International Society of Cell Therapy. Freshly isolated, uncultured cells expressed low level of mesenchymal markers such as CD105 (only 32% of cells), CD73 (47% of cells), CD90 (67% of cells), CD29 (69% of cells), and CD44 (89% of cells) and possessed antigen CD34 (over 60% of cells), HLA-DR (21% of cells), CD31 (15%), and Lin (14%) (Fig. 1). Subsequently, isolated cells were seeded into three different culture media: αMEM+10%hPL, DMEM+10%hPL, and DMEM+20%FBS+bFGF. Morphologically, plastic adherent cells, cultured in FBS-supplemented medium revealed more flattened shapes, whereas in media supplemented with hPL, the cells were more elongated and spindle-shaped (Fig. 2).

The phenotype of cultured cells changed with time and passages. Seven to 10 days after plating, when ASCs reached 80–90% confluency (passage “0,” Fig. 1), the cells were harvested and immunophenotyped again. ASCs still possessed CD34 antigens (over 50% of cells in each culture), but the expression of all mesenchymal markers increased (over 90% of cells in each culture). At passage “1,” ASCs in each culture still expressed low level of CD34 marker (~ 5% of cells) and more than 95% of cells expressed mesenchymal markers. Starting from passage “2,” phenotype of ASCs stabilizes, exhibiting mesenchymal cells features. The unique observation was the expression of CD146 antigen in cells cultured in DMEM+20%FBS+bFGF medium. The percentage of CD146^+^ cells varied from 4.5 to 55.4% and remained at a similar level throughout cell culture.

**Fig. 3:**
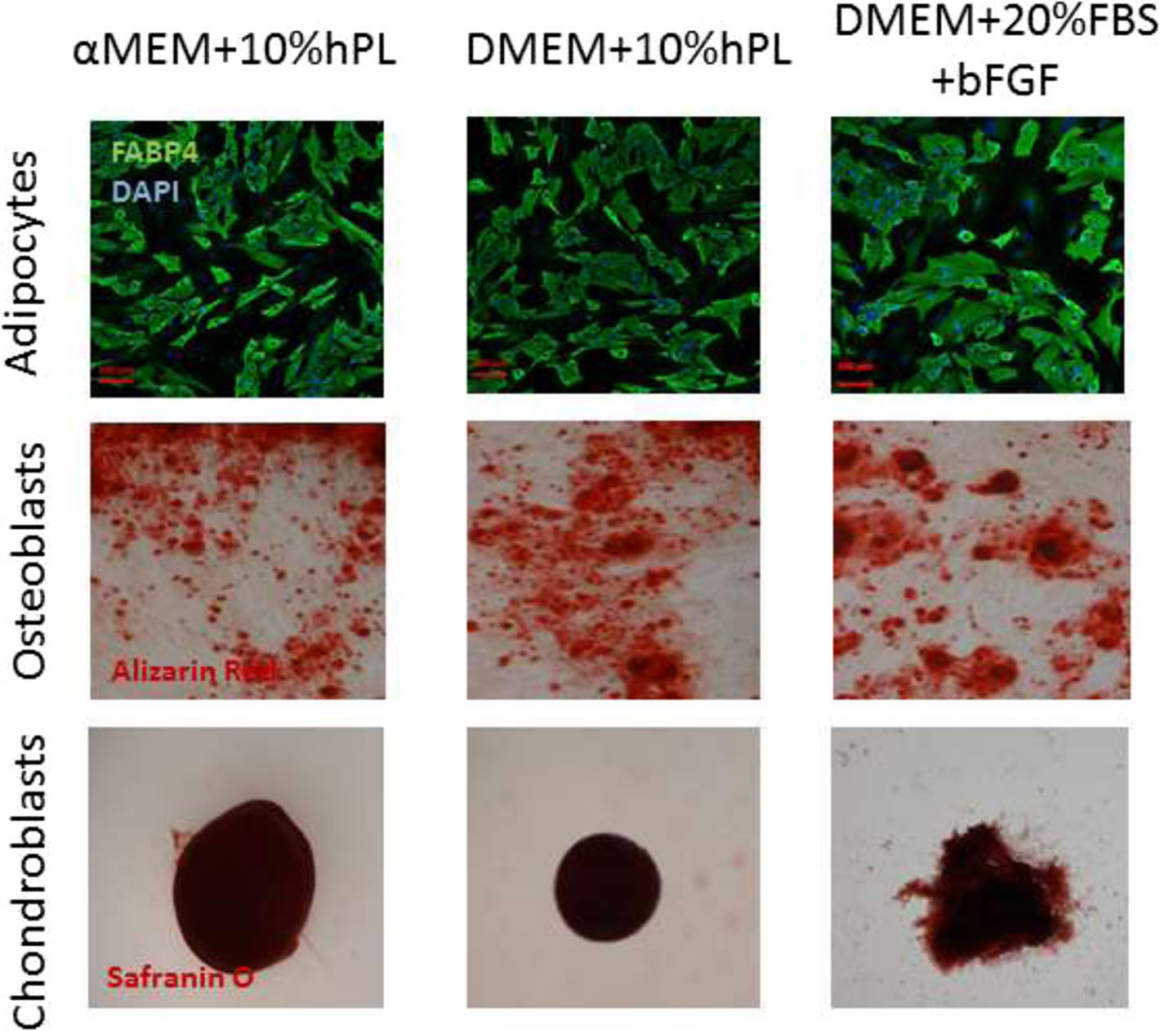
Differentiation analysis of ASCs. ASCs cultured in three different media were harvested and plated in differentiation media. ASCs differentiated in vitro into adipocytes (FABP4, green, *n* = 4, magnification × 10), osteoblasts (Alizarin Red, red, *n* = 4, magnification × 4), and chondroblasts (Safranin O, dark red, *n* = 4, magnification × 20)

The ability of ASCs cultured in three different medium formulations to differentiate into adipocytes, osteoblasts, and chondroblasts in vitro was tested on ASCs harvested from the second passage. After 21 days in adipogenic medium, characteristic lipid droplets inside the cells were observed and almost all ASCs were differentiated into adipocytes (FABP4-positive cells; Fig. 3). After 21 days in osteogenic medium, cultured cells showed typical cuboidal and flattened osteoblastic morphology and extracellular calcium deposits were stained with Alizarin Red. After 21 days in chondrogenic medium, we could detect cartilaginous proteoglycans stained by Safranin O. We did not observe differences among tested medium formulations in terms of differentiation towards adipocytes, osteocytes, and chondroblasts.

**Fig. 4:**
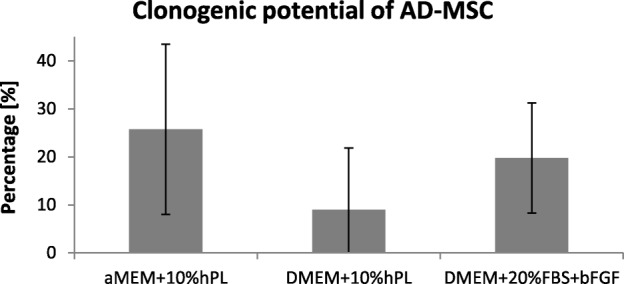
Clonogenic potential of ASCs. ASCs cultured in three different medium formulations exhibit similar clonogenic potential (*n* = 4). However statistically insignificant, more clones were obtained from single cells cultured on αMEM+10% hPL medium

In the study, we determined the clonogenic potential of ASCs cultured in three different medium formulations. The medium formulation did not influence the potential of ASCs to create clones. However statistically insignificant, the highest rate of cloning capacity showed ASCs cultured in αMEM medium supplemented with 10% hPL. On average, 26% of these cells were able to form clones (Fig. 4).

**Fig. 5:**
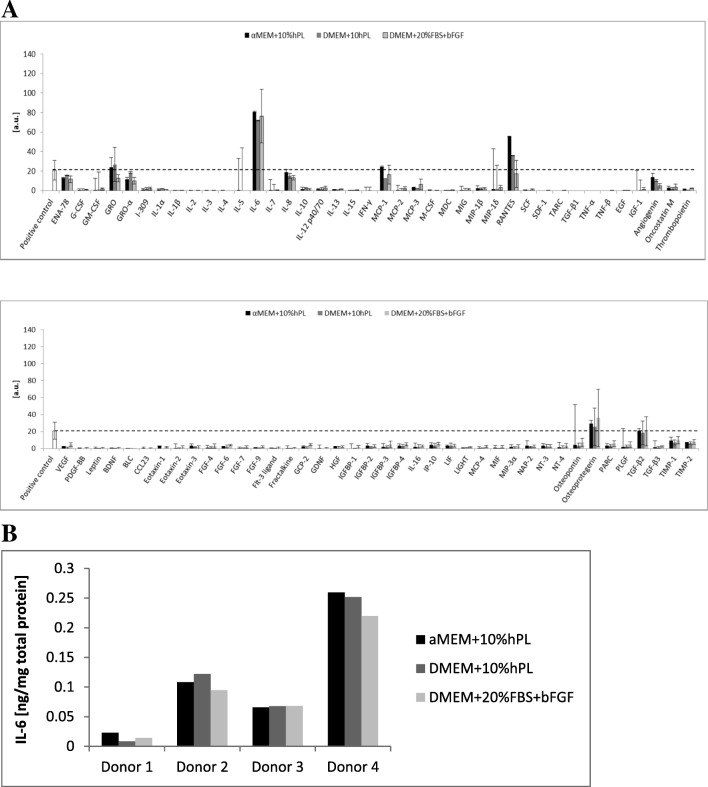
Secretome of ASCs. **a** During 48 h culture, ASCs secreted into the culture medium mainly IL-6 and small amounts of RANTES, osteoprotegerin, MCP-1, and GRO particles. The graph shows an averaged level of secreted cytokines by ASCs (*n* = 3). **b** The secretion of the most abundant cytokine—IL-6—was donor-specific, and the medium formulation did not influence its secretion. The amount of secreted IL-6 based on the ELISA assay (*n* = 4) performed in respective cell culture

ASCs cultured in three different media exhibited similar secretion profile. ASCs cultured for 48 h in media deprived of serum or platelet lysate secreted mainly interleukin 6, RANTES, osteoprotegerin, MCP-1, TGF-β2, and GRO particles (Fig. 5a). Nevertheless, IL-6 was the dominant cytokine secreted by ASCs in all culture conditions. However, the secretion of IL-6 was highly associated with a donor from whom adipose tissue was obtained. The graph (Fig. 5b) depicted the amount of secreted IL-6 in respective culture conditions depending on the tissue donor. It is clearly visible that medium formulation does not influence IL-6 secretion and is only donor-specific. Additionally, some of the ASCs did not secrete detectable amounts of IL-6 (data not shown) or secrete only a little.

**Fig. 6:**
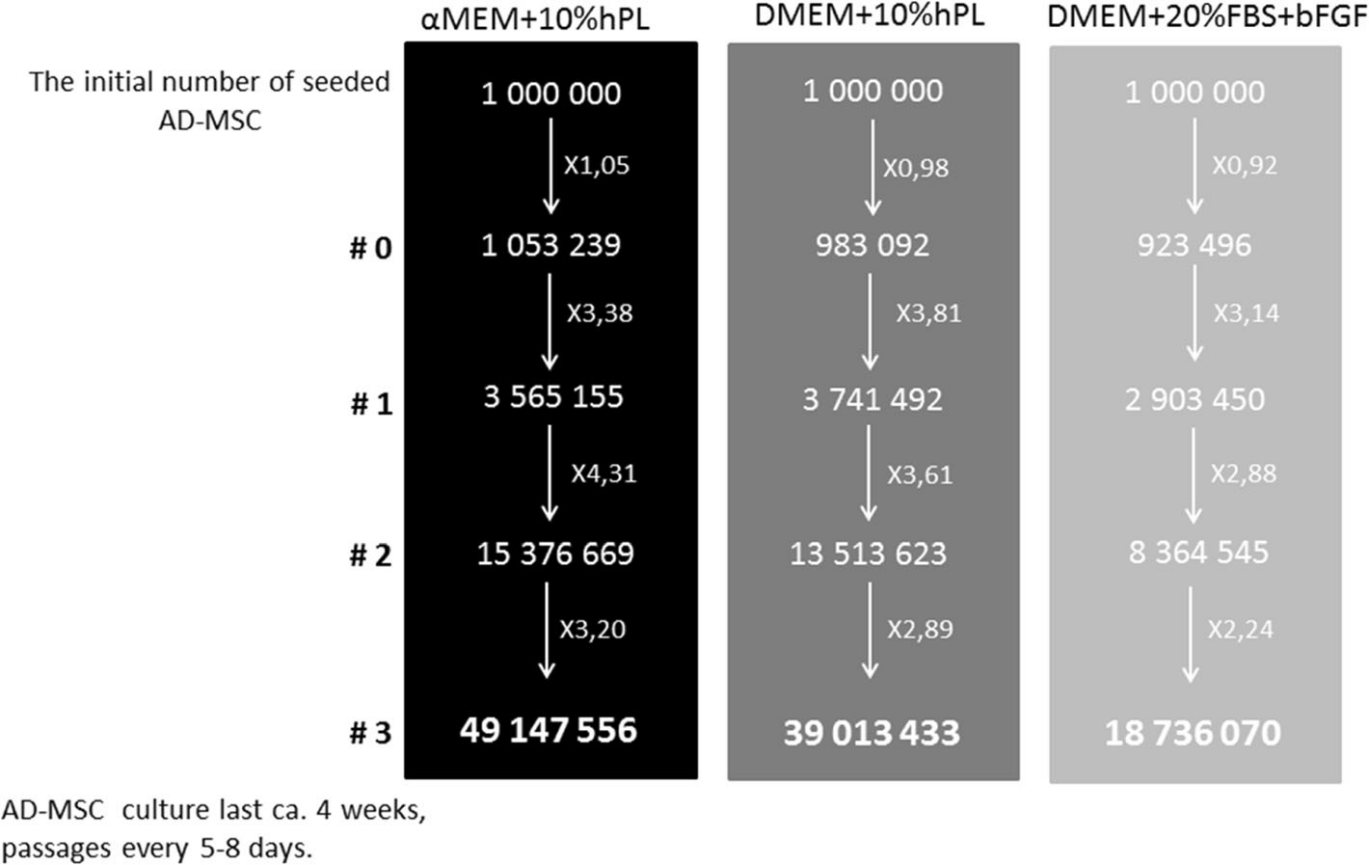
The evaluated number of cells that may be obtained in three different culture conditions. The highest yield of seeded cells may be achieved in cell culture carried out in αMEM supplemented with 10% hPL. After approximately 4 weeks, from 1 million of isolated ASCs, almost 50 million of cells may be obtained

Obtaining a sufficient number of cells adequate for cellular therapies is a crucial task in the stem cell research field. The key point is to yield a large number of cells starting with the smallest volume of tissue as possible. We evaluated the proliferation rate of cultured ASCs in three different medium formulations. Based on the number of cells obtained during cell cultures, from the initial number of one million seeded ASCs—it is approximately 2 ml of adipose tissue—we may obtain almost 50 million of ASCs in αMEM medium supplemented with 10% hPL within approximately 4 weeks. Indeed, the highest proliferation rate was observed in αMEM medium supplemented with 10% hPL, lower was in DMEM medium supplemented with 10% hPL, and the lowest in DMEM supplemented with 20% FBS and bFGF (Fig. 6). Interestingly, the proliferation rate in each culture condition was similar in the first passages; just in passage “2” the proliferation accelerated in αMEM+10% hPL medium and consequently the number of yield ASCs was the highest.

## Discussion

In this work, we have tested three different medium formulations for adipose-derived mesenchymal stromal cell (ASCs) culture. The first one consisted of α-modified Minimum Essential Medium (αMEM) as the basal medium supplemented with 10% human platelet lysate (hPL). Basal medium and hPL supplementation were obtained from Macopharma® who developed clinical-grade serum-free and xeno-free culture media dedicated to specific stem cell populations such as MSC. GMP-grade hPL is required for expanding cells for clinical use [13]. Further, hPL is currently the most promising supplement replacing FBS [13, 14]. The second medium formulation consisted of Dulbecco’s modified Eagle’s medium (DMEM) supplemented with 10% hPL. The last tested medium contained DMEM supplemented with 20% FBS and 10 ng/ml bFGF [12]. The above basal media are the most often used for MSC culture [15–19]. It was also shown that culture media based on αMEM are more suitable for both isolation and expansion of MSC [20]. We aimed to select the most effective medium formulation for ASCs expansion. Additionally, we intended to check ASCs growth condition in xeno-free culture media in order to obtain a large number of clinical-grade ASCs. FBS is typically used to expand MSC, and there were conducted successful clinical trials involving MSC expanded in FBS-containing media [14] although it is critically rated by European Medicines Agency [14]. There are severe concerns about the use of FBS for clinical applications. Growth medium consisting of animal-derived serum may lead to the introduction of xenogeneic antigens with MSC transplant and host immune rejection [21–24], possible contamination with non-human pathogens (viruses, prions, mycoplasma) and endotoxins [25–27]. FBS has also lot-to-lot variability—the concentration of growth factors may differ [27]. An ideal FBS alternative for clinical GMP production should possess well-defined composition, a reduced degree of contaminants and no risk of xenogeneic compound transmission, low production costs, easy availability, and no ethical issues [17]. Human platelet lysate meets most of these requirements and additionally contains a high level of growth factors (such as bFGF, EGF, HGF, IGF-1, PDGF, TGFβ1, VEGF) [14, 28]. Existing composition variability, which is donor-related, may be reduced by pooling different donations [29]. Possible transmission of human diseases may be overcome by virus inactivation through short-wave ultraviolet light (UV-C) [30].

By now, many reports have been published evaluating the use of hPL or other xeno-free supplements for MSC ex vivo expansion according to GMP-grade protocols [31–34]. In such studies, FBS-supplemented media represent a gold standard medium for MSC culture. It has been shown that substitution of FBS by human platelet lysate increases cell proliferation without affecting MSC immunophenotype, immunomodulatory potential, differentiation potential, and relative telomere length [34]. Indeed, we observed the highest proliferation rate of ASCs cultured in αMEM supplemented with 10% hPL; the lower rate was in DMEM supplemented with 10% hPL. Hence, the substitution of FBS by hPL accelerates the production of clinical-grade MSC in therapeutically relevant numbers [35, 36]. According to our isolation and expansion procedure, in 4 weeks, 50 million of ASCs may be obtained from a small volume of adipose tissue (2 ml). It seems also important for clinical use to not affect the immunological profile of cultured MSC. We did not observe the induction of HLA-DR antigens in all culture condition, but Sotiropoulou et al. indicated that bFGF supplementation may increase HLA class I expression [20]. There are also discrepancies between researches on immunosuppressive properties of MSC cultured in hPL-supplemented media. Some indicating that hPL-supplemented media abolish their immunosuppressive features [16], and other claims that hPL maintain these immunosuppressive properties [17]. Thus, the effect of platelet lysate on immunomodulatory characteristics of MSC remains controversial and needs to be further investigated.

Since the therapeutic potential of MSC relies on their ability to secrete a variety of soluble factors [37], we evaluated ASCs secretion profile cultured in three different medium formulations. We examine a panel of 80 secreted cytokines and growth factors. ASCs secreted into the culture medium mainly IL-6 and small amounts of chemokine CCL5 (RANTES), osteoprotegerin (OPG), MCP-1, and GRO particles. Secretion profile was unaffected by culture conditions. The most abundant cytokine was IL-6. We described earlier that MSC which secreted IL-6 through macrophages M1 ➔ M2 polarization facilitated fast functional recovery of ischemic limb and post-infarcted heart and promoted angiogenesis [12, 38]. Further, OPG cytokine (a member of the tumor necrosis factor receptor (TNFR) superfamily) stimulates the secretion of IL-6 cytokine [39].GRO particles are associated with angiogenesis promotion [40]. Therefore, secreted cytokines stimulate mainly the formation of reparative M2 macrophages and angiogenesis. However, the observed ASCs secretion profile was different than described by others [41]. It was presented that ASCs secrete mainly proangiogenic or immunomodulatory factors, such as VEGF, HGF, TGFβ, PGE2, and bFGF [42, 43]. Thus, recently, MSC has gained another moniker: medicinal signaling cells [44]. According to Caplan, medicinal signaling cells home to the sites of injured tissues and secrete factors that modulate the immune response, reduce inflammation, promote wound healing, and inhibit cell death [44, 45].

We and others [46] have shown that cytokine and growth factor secretion is donor-specific. The level of secreted IL-6 in some cases was almost at the limit of detection or even beyond, in the other was much higher. Additionally, Serra et al. have shown that cellular passaging does not influence significantly ASCs secretome properties [47]. It seems that the donor of the tissue, tissue collection method, and what is more isolation and expansion protocols influence secretion and transcription profile of isolated MSC, causing a difficult issue to compare the results between different laboratories. Additionally, Stępniewski et al. recently revealed that cells expressing the same mesenchymal markers and isolated from human heart tissue (right ventricle and epicardial fat) have distinct transcriptomes [10]. Taken together our observations concerning donor-specific secretion profile, further treatments based on mesenchymal cells need to be reevaluated.

Subcutaneous fat has become an alternative tissue source for stromal cells for regenerative medicine [48]. Adipose-derived cell therapy has shown potential in almost every preclinical animal model. It resulted in a robust clinical application often without adequate analysis of ASCs properties [6, 9]. US National Library of Medicine (clinicaltrials.gov) identified ongoing or completed 242 clinical trials with keywords “adipose derived stem cells” (905 studies found for: “mesenchymal stem cell”). To date, the majority of these clinical trials are at early phase 1 and phase 1 (only 4 trails currently at phase 4). Adipose-derived cell therapy has so far shown a favorable safety profile, but the safety assessment description has been of poor quality [49]. Safety concerns include the risk of thromboembolic complications, the use of allogeneic cells and possible rejection, and in the setting of previous cancer therapy. Subsequently, most of the studies may be classified as levels 3–4 based on the criteria according to the Center for Evidenced Based Medicine (poorly controlled case series—individual case-controlled study). In general, it is recognized as low-quality studies with increased risk of bias, which leads to an increasing chance of findings that do not represent reality [49, 50]. Therefore, it is important to design well-conducted randomized controlled trials with adequate blinding, including a placebo/sham treatment, and the safety assessment together with the application of clinical-grade ASCs [51].

## Conclusions

Taken together, our results indicate that xeno-free media do not alter the typical for mesenchymal cell expression pattern, multipotentiality, and secretion profile. However, the proliferation rate is higher when compared with conventionally used medium formulation containing xenogeneic serum. Thus, the media containing hPL as a serum xeno-free substitute are suitable for clinical use when in a relatively short time, the large-scale expansion is required.

Nevertheless, there is a great need to improve the understanding of the biology of cells currently termed MSC. The main concern is the severe difference in the gene expression, transcription and secretion profile, and differentiation potential between cells isolated from different tissues, different donors, or even different regions of the same tissue. Still, despite their heterogeneity, indisputably is their ability to secrete cocktails of proteins that modulate the immune response, reduce inflammation, promote wound healing, and inhibit cell death. What we need to do is to resolve the true nature of MSC and conditions which will allow to culture MSC as a precise and established cell product suitable for clinical use.

## Data Availability

Not applicable.

## Abbreviations

ASCs: Adipose tissue-derived mesenchymal stromal cells
ATMP: Advanced therapy medicinal products
bFGF: Basic fibroblast growth factor
DMEM: Dulbecco’s modified Eagle’s medium
FABP4: Fatty acid-binding protein 4
FBS: Fetal bovine serum
GMP: Good Manufacturing Practice
GRO: Growth-regulated protein
hPL: Human platelet lysate
IL-6: Interleukin 6
ISCT: International Society for Cell Therapy
MCP-1: Monocyte chemoattractant protein-1
PBS: Phosphate-buffered saline
RANTES: Regulated on Activation, Normal T cell Expressed and Secreted
TGF-β2: Transforming growth factor beta 2
αMEM: Minimum Essential Medium Eagle Alpha Modification
